# Pneumatosis intestinalis in abdominal CT: predictors of short-term mortality in patients with clinical suspicion of mesenteric ischemia

**DOI:** 10.1007/s00261-022-03410-x

**Published:** 2022-01-20

**Authors:** Simon D. Graber, Stefanie Sinz, Matthias Turina, Hatem Alkadhi

**Affiliations:** 1 Institute of Diagnostic and Interventional Radiology, University Hospital Zurich, University of Zurich, Raemistr. 100, 8091 Zürich, Switzerland; 2 Department of Visceral Surgery, University Hospital Zurich, University of Zurich, Zürich, Switzerland

**Keywords:** Mesenteric ischemia, Pneumatosis intestinalis, Mortality, Computed tomography

## Abstract

**Purpose:**

Pneumatosis intestinalis (PI) in the bowel wall demonstrated in computed tomography (CT) of the abdomen is unspecific and its prognostic relevance remains poorly understood. The purpose of this study was to identify predictors of short-term mortality in patients with suspected mesenteric ischemia who were referred to abdominal CT and showed PI.

**Methods:**

In this retrospective, IRB-approved, single-centre study, CT scans and electronic medical records of 540 patients who were referred to abdominal CT with clinical suspicion of mesenteric ischemia were analysed. 109/540 (20%) patients (median age 66 years, 39 females) showed PI. CT findings were correlated with surgical and pathology reports (if available), with clinical and laboratory findings, and with patient history. Short-term outcome was defined as survival within 30 days after CT.

**Results:**

PI was found in the stomach (*n* = 6), small bowel (*n* = 65), and colon (*n* = 85). Further gas was found in mesenteric (*n* = 54), portal (*n* = 19) and intrahepatic veins (*n* = 36). Multivariate analysis revealed that PI in the colon [odds ratio (OR) 2.86], elevated blood AST levels (OR 3.00), and presence of perfusion inhomogeneities in other abdominal organs (OR 3.38) were independent predictors of short-term mortality. Surgery had a positive effect on mortality (88% lower likelihood of mortality), similar to the presence of abdominal pain (65% lower likelihood).

**Conclusions:**

Our study suggests that in patients referred for abdominal CT with clinical suspicion of mesenteric ischemia, location of PI in the colon, elevation of blood AST, and presence of perfusion inhomogeneities in parenchymatous organs are predictors of short-term mortality.

**Graphical abstract:**

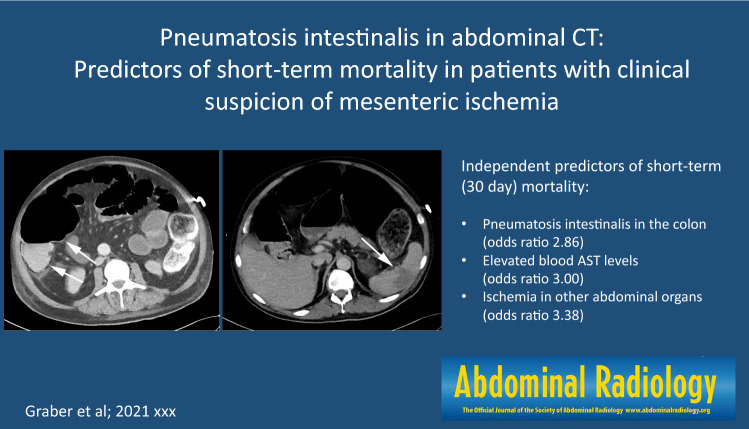

## Introduction

Pneumatosis intestinalis (PI) refers to the presence of gas within the wall of the small or large intestine, which is usually diagnosed with abdominal computed tomography (CT). CT typically demonstrates a low density linear and/or bubbly pattern of gas within the bowel wall, which can be readily diagnosed with CT due to its high spatial resolution and multiplanar reformation capabilities [1–3].

The pathogenesis of PI remains poorly understood and is probably multifactorial. PI may be an incidental finding associated with a benign etiology, whereas in others, it portends a life-threatening abdominal condition. Therefore, the significance of PI usually depends on the nature and severity of the underlying etiology [4]. One of the most feared underlying conditions for PI is mesenteric ischemia, which is associated with a high morbidity and mortality [5]. Because of the wide array of clinical settings in which PI is encountered, it is recommended to interpret radiological findings together with the individual clinical presentation, laboratory findings and patient history, in order to ensure a correct diagnosis and to guide appropriate management [6].

Despite of this, the implications of PI are often misinterpreted and patients are being operated for suspected mesenteric ischemia, finally demonstrating normal, non-ischemic bowel during surgery. While some authors suggested that PI, particularly when combined with portal-venous gas, was associated with mesenteric ischemia in 69% of patients and with a mortality of 50% [7], others showed that 40% of patients with PI on CT and clinical findings being suggestive of mesenteric ischemia had viable bowel at surgery or recovered without surgery [8]. The latter finding was further corroborated by Wiesner et al. [9] who showed that PI on CT does not always indicate transmural necrosis of the bowel wall. Also, the prognostic implications of PI seen in abdominal CT remains poorly understood.

The purpose of our study was to identify predictors of mortality in patients admitted to abdominal CT with suspicion of mesenteric ischemia and showing PI of the bowel wall.

## Materials and methods

### Study population

In this retrospective study, we searched in our electronic database system in the years from January 2014 to December 2019 for the terms “mesenteric ischemia”, “bowel ischemia” and “intestinal ischemia” in all radiological reports of patients who underwent a non-enhanced and contrast-enhanced abdominal CT examination in the arterial and portal-venous phase. The entire report text was screened for the terms mentioned above. The clinical suspicion of mesenteric ischemia was taken from the order indication.

We found a total of 540 patients. 408/540 patients (76%) had no PI in CT and were thus excluded. Care was taken to not mistake pseudo-pneumatosis as PI [10]. Twenty-three of the remaining 132 patients (30%) had to be excluded because of a missing signed consent. The remaining 109 patients were included in this study (Table 1). This study had institutional review board and local ethics committee approval.

**Table 1:** Patient demographics

	Surgery	Conservative management	Overall
	*n* = 85	*n* = 24	*n* = 109
Gender			
Female	30 (28%)	9 (8%)	39 (36%)
Male	55 (51%)	15 (14%)	70 (64%)
Age (years)			
Mean (SD)	66 (± 15)	62 (± 14)	65 (± 14)
Median	66	63.5	66
CT scan range			
Abdomen	46 (42%)	9 (8%)	55 (51%)
Chest abdomen	32 (29%)	15 (14%)	47 (43%)
Neck chest abdomen	4 (4%)	0	4 (4%)
Died during index hospitalization			
Yes	49 (45%)	22 (20%)	71 (65%)
No	36 (33%)	2 (2%)	38 (35%)
Smoking			
Yes	55 (51%)	13 (12%)	68 (62%)
No	25 (23%)	11 (10%)	36 (33%)
Diabetes			
Yes	26 (24%)	5 (5%)	31 (28%)
No	59 (54%)	18 (17%)	77 (71%)
Obesity			
Yes	36 (33%)	12 (11%)	48 (44%)
No	47 (43%)	11 (10%)	58 (53%)
Nausea			
Yes	29 (27%)	7 (6%)	36 (33%)
No	55 (51%)	16 (15%)	71 (65%)
Vomiting			
Yes	38 (35%)	10 (9%)	48 (44%)
No	46 (42%)	13 (12%)	59 (54%)
Abdominal pain			
Yes	58 (53%)	10 (9%)	68 (62%)
No	26 (24%)	13 (12%)	39 (36%)

### CT imaging

All CT examinations were performed on a second or third generation dual-source CT scanner (SOMATOM Definition Flash or Force, Siemens Healthineers, Forchheim, Germany) using our institutional multiphasic abdominal CT protocol settings including a non-enhanced, followed by an arterial and portal-venous phase CT scan (intravenous administration of 70–100 mL iodinated contrast media depending on the body weight). Standard image reconstructions were performed with a slice thickness of 2 mm (increment 1.5 mm) using a medium-soft tissue convolution kernel.

### Image interpretation

Two radiologists (one with 4 years and one with 15 years of experience in abdominal imaging) being blinded to the clinical data evaluated independently all abdominal CT examinations. The two readers evaluated all imaging findings being indicative of mesenteric ischemia listed in Table 2 [11]. Readers were requested to note the anatomical location of all abnormal findings. Concerning the bowel wall, the criteria included wall thickening (> 0.5 cm), non-enhanced hyperdense wall, distension of the bowel (defined as > 3 cm in small bowel, > 9 cm in cecum and > 6 cm in colon), reduced wall enhancement, and presence of ileus (mechanical vs paralytical). For the abdominal cavity, the readers listed the presence of mesenteric edema (*misty/dirty* mesenteric fat), ascites (the presence of fluid in anatomical pouches), free air and additional locations demonstrating ischemia (namely the liver, spleen, kidneys) [12]. The abdominal arteries and veins were evaluated for atherosclerosis and occlusion. In case of disagreement, consensus reading of the two radiologists was performed, which was required in 11 of the 109 patients (10%).

**Table 2:** Extracted data from abdominal CT and from the electronic patient records

CT findings	Pneumatosis intestinalis location	Stomach
		Small bowel
		Cecum
		Colon
		Sigma
		Mesenteric
		Portal
		Intrahepatic
	Bowel	Wall thickening
		Hyperdense wall on non-enhanced phase
		Distension
		Reduced enhancement of bowel wall
		Ileus (mechanical, paralytic)
	Abdominal cavity	Mesenteric edema
		Ascites
		Additional perfusion inhomogeneities
		Free air
	Vessels	Atherosclerosis
		Vascular occlusion (arterial/venous)
Laboratory tests	Liver and Pancreas	LDH^a^ normal values 50–150 g/L
		AST^b^ normal values 7–37 IU/L
		ALT^c^ normal values 7–56 U/L
		ALP^d^ normal values 48–113 U/L
		*Lipase*
		*Pancreatic amylase*
	Kidney	Urea normal values 3–7 mmol/L
		Creatinine normal values 60–108 mmol/L
	Heart	Ck total^e^ normal values 38–260 U/L
		Myoglobin normal values 9–86 ng/mL
	Miscellaneous	Phosphate normal values 0.8–1.5 mmol/L
		Bicarbonate normal values 18–23 mmol/L
		pH^f^ normal values 7.34–7.44
		Lactate normal values 0.5–2.2 mmol/L
	Inflammatory	CRP^g^ normal values 0–5 mg/L
		*PCT*^h^
	Blood findings	HB^i^ normal values 120–160 g/L
		Leukocytes normal values 4–10 G/L
		Albumin normal values 35–55 g/L
Clinical findings	Symptoms	Nausea
		Vomiting
		Abdominal pain
	Vital parameters	Pulse
		Blood pressure (systolic/diastolic)
		Fever
Patient history	Cardiovascular risk factors	Smoking
		Diabetes
		Obesity
		BMI^j^
		PAOD^k^
		Heart disease
		Atrial fibrillation
		Additional surgery
Treatment	Conservative	
	Surgery	Exploratory (laparotomy and laparoscopy)
		Curative
Histopathology	Not available	
	No abnormality	
	Ischemia	
	Infection	
	Inflammation	

Cursive written parameters were later excluded by the reason of the frequency of appearance

^a^*LDH* lactate dehydrogenase

^b^*AST* aspartate aminotransferase

^c^*ALT* alanine aminotransferase

^d^*ALP* alkaline phosphatase

^e^Ck total creatine kinase

^f^*pH* potential of hydrogen

^g^*CRP* C-reactive protein

^h^*PCT* procalcitonin

^i^*HB* hemoglobin

^j^*BMI* body mass index

^k^*PAOD* peripheral arterial occlusive disease

### Data analysis

One of the readers (with 4 years of experience) collected laboratory tests, clinical findings, patient history, treatment during hospitalization and results from surgery and histopathology, if available, for all patients (see Table 2). The reviewed variables included also age, sex, smoking, obesity, body mass index (BMI), comorbidities such as diabetes and heart disease (including coronary atherosclerosis, heart failure such as hypertensive cardiomyopathy, valvular stenosis, previous myocardial infarction, heart transplantation), other risk factors such as underlying malignancy, as well as symptoms (pain, nausea, vomiting), and clinical findings at the time of presentation and CT.

### Treatment and patient outcome

The 109 patients were divided into two groups: Those who were operated (*n* = 85/78%) and those who were not operated within 30 days of CT (*n* = 24/22%). Patients who were not operated underwent conservative therapy according to the underlying disease.

**Fig. 1:**
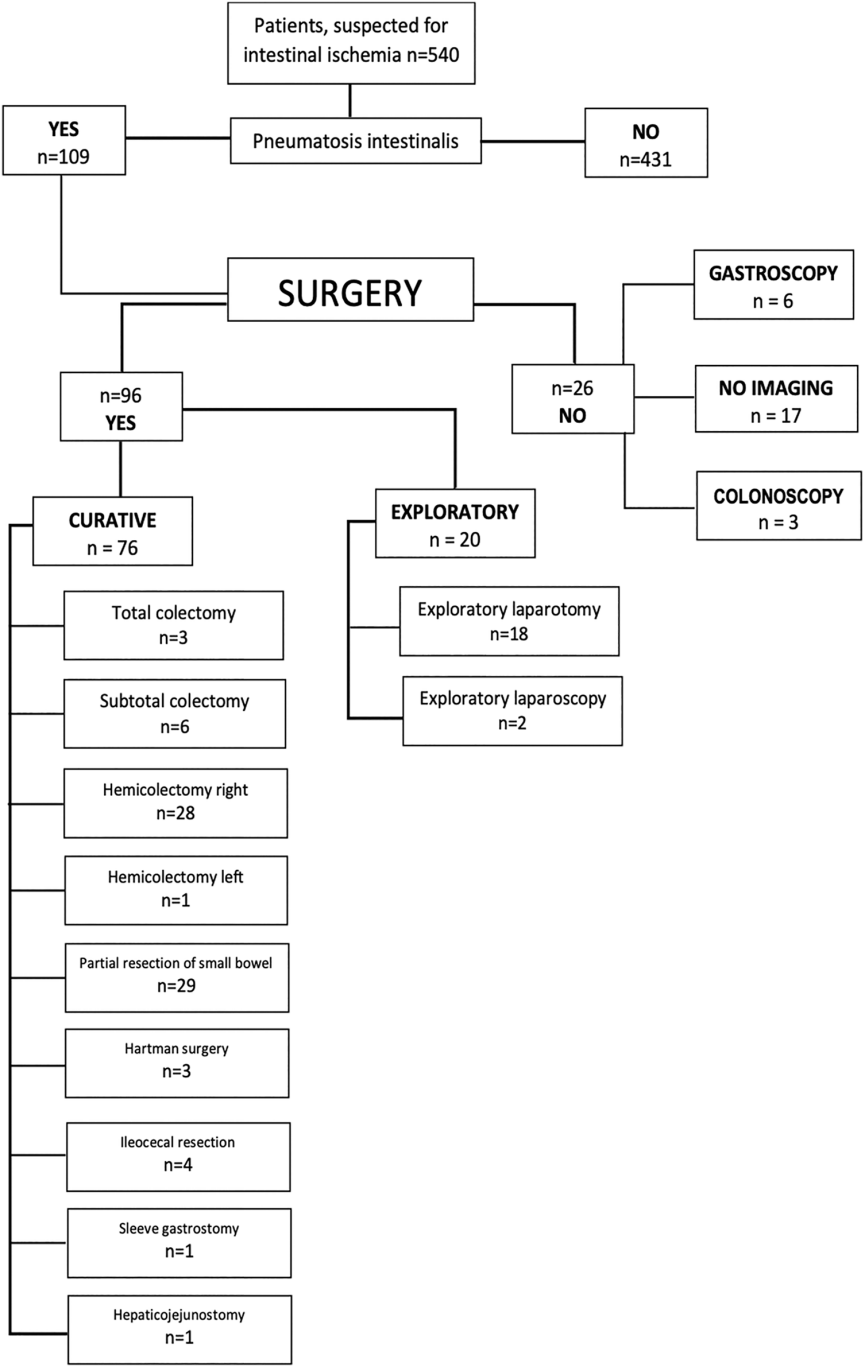
Study flowchart. Please note that the total amount exceeds the number of patients as some received both endoscopy and surgery

Patients who underwent surgery were divided into a group with curative intent and to an exploratory group (which are not mutually exclusive, as there were explorative laparotomies that turned into surgery with curative intent, Fig. 1). Laparotomy and laparoscopy were categorised in the explorative group. The following types of surgery (i.e. subtotal and total colectomy, hemicolectomy right and left, partial resection of the small bowel, Hartmann surgery, ileocecal resection, sleeve gastrostomy and hepaticojejunostomy) were noted. Conservative treatment included, among others, the application of antibiotics, bowel rest and/or palliative care.

Mortality rates were calculated for 30 days after CT (hereafter called short-term mortality).

### Statistical analysis

A binary regression model was conducted to predict short-term mortality of the present data. A total of 78 bivariate models were constructed, one for each predictor variable of interest. Mortality was coded as a binary variable representing death, while the predictors were coded as continuous, ordinary (categories), nominal (each category regarded separately such as normal, high or low levels), and binary variables.

There were nine possible groups of correlated variables and one such group was discovered in the data (nausea and vomiting). Multi-collinearity tests, tests for suppression effects and confounding variables were performed to distinguish the independent effect of each predictor. In the cases where the variables were highly correlated, a factor score encompassing all of them was constructed after a parallel analysis to determine the number of components. Additionally, a principal component analysis was performed to calculate the respective score. This factor score was then used to calculate the unbiased effect of the variables.

Four separate multivariate models were conducted with the location of pneumatosis, distension, thickening, and edematous bowel wall as predictors. One multivariate logistic regression was conducted with these abnormalities in the colon as predictors. This was done because their effects were both significant in the previously calculated bivariate and multivariate models and there was a possibility of confounding effects. Finally, a multivariate model was conducted by including all significant variables. After tests of multicollinearity, likelihood ratio tests, and comparisons of information criteria, a model with an optimal set of predictors was constructed. All statistical analyses were performed using commercially available statistical software (R, R Core Team, version 4.0.2).

## Results

### Patient characteristics

The mean age of the 109 patients referred to imaging with a clinical suspicion of mesenteric ischemia and PI on abdominal CT was 66 ± 15 years. There were 39 females (36%) and 70 males (64%). The majority of patients (90/109, 83%) were in patients. The suspected etiology of PI was bowel obstruction in most patients (43/109, 39%), followed by vessel occlusion (30/109, 28%). The third most commonly suspected etiology was carcinoma in 18 patients (17%), while more rare probable causes were: pancreatitis, *n* = 4; trauma, *n* = 2; colitis, *n *= 1; Crohn’s disease, *n* = 1; and lymphoma, *n *= 1 (Table 3). The etiology of PI remained unknown in nine patients (10%). Of the 109 patients with PI, 66 patients (61%) had histopathological proof of mesenteric ischemia.

**Table 3:** Possible underlying etiology of PI in the patient population

	Number of patients (%)	Number of deceased patients	Mortality rate in %
Bowel obstruction/ileus	43 (39%)	37	86
Vascular occlusion	30 (28%)	23	77
Carcinoma	18 (17%)	5	28
Unknown	9 (8%)	6	67
Pancreatitis	4 (4%)	0	0
Trauma	2 (2%)	2	100
Clostridium difficile colitis	1 (1%)	0	0
Crohn’s disease	1 (1%)	0	0
Lymphoma	1 (1%)	0	0

### Treatment during index hospitalization

As shown in Fig. 1, treatment information did not correspond with the number of patients. In the conservatively managed group, 17 of 24 patients did not undergo additional procedures, while the other 7 patients received one or two endoscopies (colonoscopy and/or gastroscopy). In the surgical patient group, the total number of procedures was 96, with a total number of 76 (79%) curative procedures.

### Mortality

Overall, 71 of 109 patients died within one month, indicating a cumulative probability of short-term mortality of patients with clinical suspicion of mesenteric ischemia and PI on CT of 65%. In total, 24 of the 109 patients (22%) underwent conservative treatment, from which 22 died within one month, which indicates a 92% mortality in the conservative treatment group. 85 (78%) of the patients underwent surgery indicating a 58% mortality rate in the surgery group.

### Clinical, laboratory and CT imaging variables

Elevated blood levels of aspartate aminotransferase (AST) were found in 69 of the 109 patients (63%). The same number of patients (69/109, 63%) had elevated blood lactate levels. A decreased blood pH was found in 59/109 patients (54%). AST and blood lactate levels showed a negative and significant correlation with short-term mortality (*p* < 0.01), and blood pH levels had a negative correlation with short-term mortality (*p* < 0.01). Presence of abdominal pain (68/109 patients, 62%) correlated positively with mortality (*p* < 0.02).

Bivariate analysis showed significant differences of the parameters abdominal pain, surgery, elevated blood levels of AST and lactate, and lower levels of pH between patients who died and those who did not (all, *p* < 0.05).

In regard to CT, the presence of PI in the colon, reduced enhancement of the colon wall, distension of the colon, and ischemia of other abdominal organs showed significant differences between groups (all, *p* < 0.05) (Table 4).

**Table 4:** Results from bivariate analysis

Variable	Number of patients	Mortality (numbers)	Mortality (%)	Bivariate analysis		*p* value
				Odds ratio	95% Confidence interval	
Operation						
No	24	22	92	–	–	–
Yes	85	49	58	0.12	0.02–0.46	< 0.01
AST						
Normal	30	13	43	–	–	–
High	69	53	77	4.33	1.76–11.05	< 0.01
pH						
Normal	37	19	51	–	–	–
High	12	5	42	0.68	0.17–2.51	0.56
Low	59	47	80	3.71	1.52–9.38	< 0.01
Lactate						
Normal	37	18	49	–	–	–
High	69	51	74	2.99	1.30–7.02	0.01
Low	1	0	0	–	–	–
Pain						
No	39	31	79	–	–	–
Yes	68	39	57	0.35	0.13–0.84	0.02
Pneumatosis (colon)						
No	62	34	55	–	–	–
Yes	47	37	79	3.05	1.32–7.46	0.01
Distension (colon)						
No	82	49	60	–	–	–
Yes	27	22	81	2.96	1.09–9.55	0.05
Reduced enhancement of bowel wall (colon)						
No	87	52	60	–	–	–
Yes	22	19	86	4.26	1.33–19.11	0.03
Ischemia in other abdominal organs additional organs						
No	84	49	58	–	–	–
Yes	25	22	88	5.23	1.65–23.34	0.01

The two CT findings that correlated with short-term mortality were location of PI in the colon, which was observed in 47 (43%) of the patients and showed a mortality rate of 79%. The second CT finding was presence of perfusion inhomogeneities in parenchymatous abdominal organs (i.e., spleen, liver, kidneys), which was found in 25 (23%) of the patients and which was associated with a substantial mortality (88%) (Table 4).

The three additional CT findings showing some association with short-term mortality were mesenteric edema (*n* = 90, 83%, *p* = 0.08), ascites (*n* = 80, 73%, *p* = 0.08), and distension of the colon (*n* = 22, 20%, *p* = 0.05). However, these parameters did not reach statistical significance.

### Independent predictors of short-term mortality

**Fig. 2:**
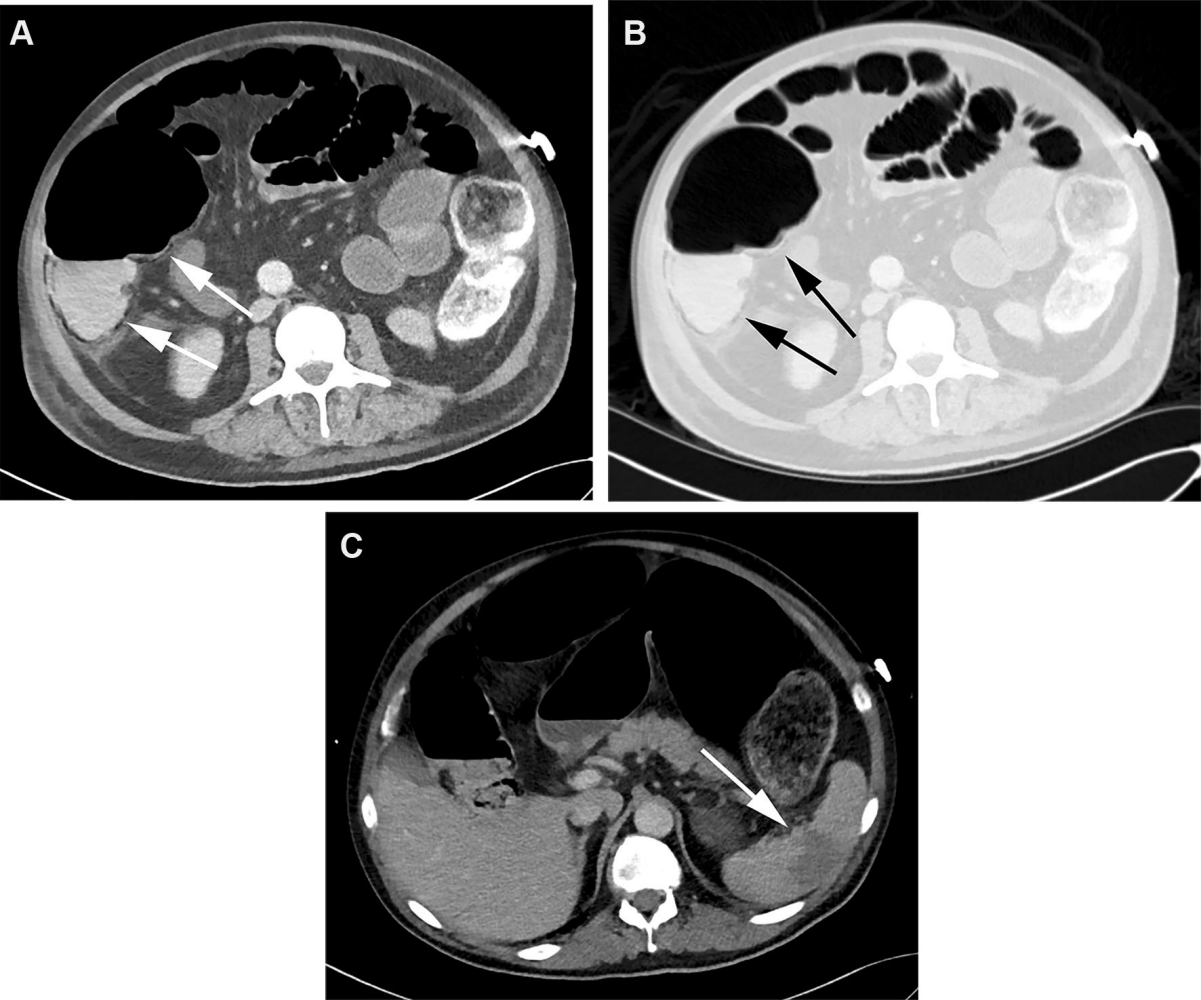
CT in the soft tissue (**a**) and lung window (**b**) of a 70-year-old male patient with PI in the colon (arrows) and distension of the small bowel and colon. In addition, perfusion inhomogeneities indicating ischemia in the spleen were found (**c**, arrow). The AST blood level was 80 IU/L (normal values 7–37 IU/L), the blood lactate level was 8.1 mmol/L (normal values 0.5–2.2 mmol/L). The patient died one day after CT

**Fig. 3:**
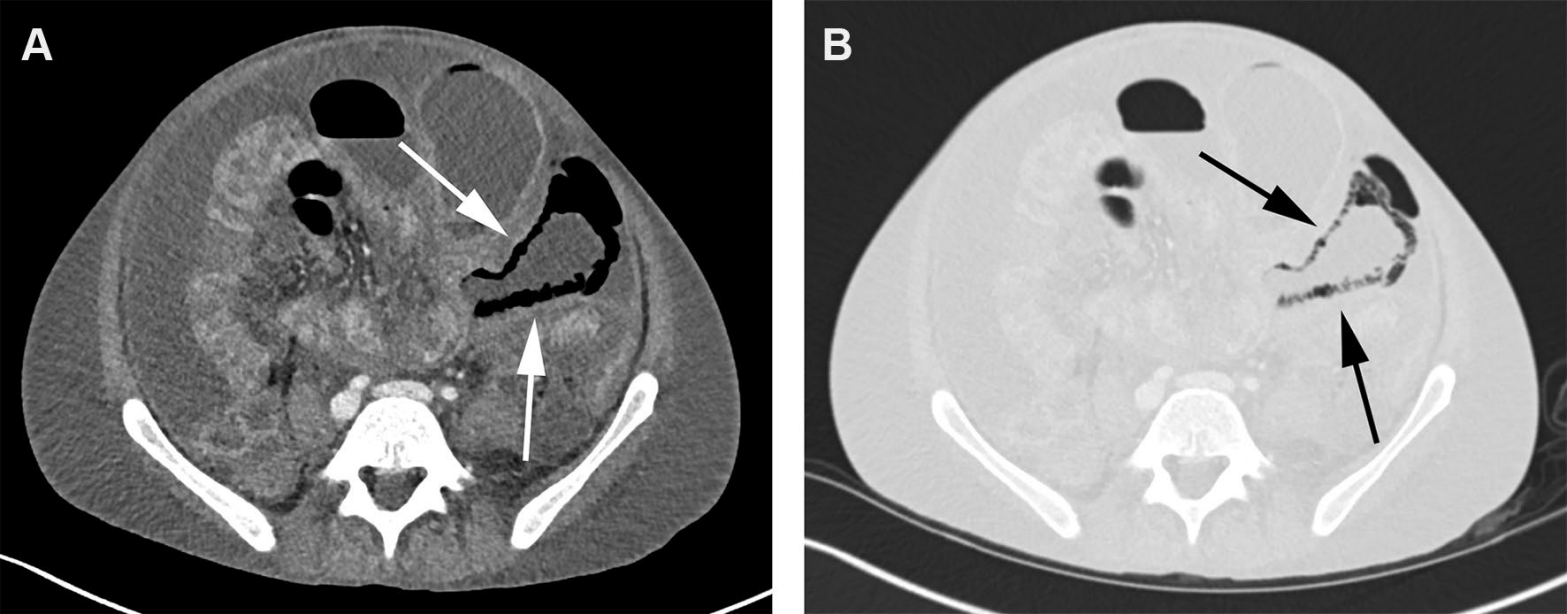
CT in the soft tissue (**a**) and lung window (**b**) of a 29-year-old male patient with PI in the small bowel (arrows) as well as gas in the mesenteric veins (not shown). The small bowel was distended and showed wall thickening as well. Ascites was present. The AST blood level was 18 IU/L (normal values 7–37 IU/L), the blood lactate level was 3.1 mmol/L (normal values 0.5–2.2 mmol/L). The patient recovered completely

Multivariate analysis revealed that the parameters PI in the colon (odds ratio (OR) 2.86, 95% CI 1.08–8.04), surgery (OR 0.16, 95% CI 0.02–0.64), and elevated AST blood levels (OR 3.00, 95% CI 1.10–8.44) were significant, independent predictors of short-term mortality (Table 5). Presence of perfusion inhomogeneities in other abdominal organs had an OR of 3.38 (95% CI 0.96–16.01), however, did not reach the statistical level of significance (*p* = 0.08). Kaplan Meier analysis showed the difference of mortality between patients with elevation of AST versus those with normal AST blood values. Surgery had a positive effect on mortality (88% lower likelihood of mortality), similar to the presence of abdominal pain (65% lower likelihood). Representative image examples are provided in Figs. 2 and 3.

**Table 5:** Results from multivariate analysis

Variable	Number of patients	Mortality (numbers)	Mortality (%)	Multivariate analysis		*p* value
				Odds ratio	95% Confidence interval	
Pneumatosis (Colon)						
No	62	34	55	–	–	
Yes	47	37	79	2.86	1.08–8.04	0.04
Surgery						
No	24	22	92	–	–	
Yes	85	49	58	0.16	0.02–0.64	0.02
AST						
Normal	30	13	43	–	–	
High	69	53	77	3.00	1.10–8.44	0.03
Ischemia in other abdominal organs						
No	84	49	58	–	–	
Yes	25	22	88	3.38	0.96–16.01	0.08

## Discussion

PI is a frequent finding in abdominal CT, but its presence is rather unspecific [1–3] and the prognostic value of this sign on CT is not yet clearly defined. Our study evaluated predictors of short-term mortality in patients in whom there was a clinical suspicion of mesenteric ischemia and in whom PI was found in CT. Results of this study suggest that the localization of PI in the colon, elevation of blood AST levels, and the presence of perfusion inhomogeneities in parenchymatous abdominal organs such as the liver and spleen were independent predictors of short-term mortality in these patients.

The clinical and imaging diagnosis of mesenteric ischemia remains challenging. Particularly the finding of PI, when not taking into account also other CT signs of mesenteric ischemia, is neither sensitive nor specific [11, 13–15]. This is well reflected by the results of our study in which 39% of patients with a clinical suspicion of mesenteric ischemia and who had PI in the bowel wall had no histopathological proof of mesenteric ischemia.

The high utilization rate of CT imaging in patients with suspected mesenteric ischemia and, along with this, the frequent finding of PI causes a dilemma, since the presence of PI alone is not helpful in guiding further treatment [1–4]. Greenstein et al. [16] and Wayne et al. [17] proposed management algorithms for patients with PI having the aim to divide patients into those requiring surgery and those who do not. Both these studies suggested using PI combed with clinical evaluation and laboratory findings. Greenstein et al. [16] found that patients with PI, being older than 60 years, having a white blood cell count above 12 c/mm^3^ and/or emesis were most likely to have surgical intervention. In their study, patients with PI and sepsis had the highest risk for death. Wayne et al. [17] developed a different algorithm including a score comprising risk factors, coronary artery and peripheral vascular disease, heart failure, arrhythmia, sepsis, vasculitis or venous occlusion, abdominal pain, elevated lactate levels and small bowel PI for guiding patient management [17].

Thompson et al. [18] evaluated in animal experiments a correlation between mesenteric ischemia and various enzymes. Similar to imaging, however, these laboratory biomarkers were unspecific as well. The most commonly reported associations with mesenteric ischemia were elevated blood AST and blood lactate levels [9]. In a systematic review, Ekin et al. [19] analyzed the relationship between lactate levels and mesenteric ischemia and reported a high sensitivity but low specificity, the latter often below 50%. Elevated blood lactate levels have been reported to be a late finding in the course of mesenteric ischemia not predicting mortality [19]. Some authors tried to create models including lactate levels with accompanying conditions such as sepsis [16] and metabolic acidosis [17]. However, lactate was still found to be of limited use for predicting the course and mortality in patients with mesenteric ischemia.

AST is a sensitive indicator of necrosis of various human tissues including the bowel [20]. In addition, AST has been described as an independent predictor of death in patients with acute mesenteric ischemia [21]. Still, liver enzymes such as AST show limited sensitivity (73%) and specificity (60%) [22].

Beyond the localization of PI in the colon and elevated blood AST levels, we found ischemia in other organs than the bowel such as the liver or spleen to show a trend as another predictor of short-term mortality. The latter variable, however, did not reach statistical significance, although the odds ratio was high (3.38). We assume that ischemia in parenchymal abdominal organs likely reflects advanced and more severe disease including sepsis and heart failure, which is associated with higher morbidity and mortality.

The following limitations of our study merit consideration. First, the study was retrospective and reflects a single-centre experience, which limits the generalizability of the results. Second, mainly in-house patients were included, which indicates further limited generalizability to a different population. Third, sample size was rather small. This is the most probable reason why the variable ischemia in other abdominal organs failed to reach statistical significance in multi-variate analysis. Fourth, there might have been variation in words used in the various radiological reports, which may have resulted in missed patients using our search terms. Fifth, we cannot rule-out that we misdiagnosed patients as having PI although pseudo-pneumatosis was present. However, we tried to avoid misdiagnosis using previously described criteria [10]. Sixth, we cannot rule-out milder forms of mesenteric ischemia in patients who were not operated and who have no histopathological proof of ischemia. Finally, it was not possible to determine the etiology of PI in all patients.

In conclusion, our study suggests that—in patients referred to abdominal CT with a clinical suspicion of mesenteric ischemia and who show PI—location of PI in the colon, elevated blood AST levels, and ischemia in other abdominal parenchymatous organs are independent predictors of short-term mortality.

## Data Availability

Data available on request from the authors.

## Code availability

The datasets generated during and/or analysed during the current study are available from the corresponding author on reasonable request.
